# Hepatic stroma-educated regulatory DCs suppress CD8^+^ T cell proliferation in mice

**DOI:** 10.18632/oncotarget.18459

**Published:** 2017-06-13

**Authors:** Qian Wang, Hao He, Dongwei Chen, Chao Wang, Yingping Xu, Wengang Song

**Affiliations:** ^1^ Institute of Immunology, Taishan Medical University, Taian, Shandong, China; ^2^ Center Hospital of Taian City, Taian, Shandong, China; ^3^ Institute of Immunology, School of Medicine, Tsinghua University, Beijing, China

**Keywords:** liver, regulatory dendritic cells, CD8^+^ T cells, nitric oxide, autoimmune hepatitis, Immunology and Microbiology Section, Immune response, Immunity

## Abstract

Liver dendritic cells (DCs) display immunosuppressive activities and inhibit the CD4^+^ T cell response. The present study assessed whether and how liver DCs suppress CD8^+^ T cells. We found that bone marrow-derived mature DCs incubated with liver stromal cells were characterized by a longer life span, reduced CD11c, IA/IE, CD80, CD86, and CD40 expression, and increased CD11b expression. These unique liver stromal cell-educated mature DCs (LSed-DCs) stimulated CD8^+^ T cells to express CD25 and CD69, but inhibited their proliferation. CD8^+^ T cell suppression depended on soluble factors released by LSed-DCs, but not cell-cell contact. Compared with mature DCs, LSed-DCs produced more nitric oxide and IL-10. Addition of a nitric oxide synthase inhibitor, PBIT, but not an IL-10-blocking mAb, reversed LSed-DC inhibition of CD8^+^ T cell proliferation. We also found that LSed-DCs reduced CD8^+^ T cell-mediated liver damage in a mouse model of autoimmune hepatitis. These results demonstrate that the liver stroma induces mature DCs to differentiate into regulatory DCs that suppress CD8^+^ T cell proliferation, and thus contribute to liver tolerance.

## INTRODUCTION

The liver is a lymphoid organ [1] that consists of two major lymphocyte groups: an innate component including abundant NK cells and NKT cells, and an adaptive component containing conventional CD4^+^ and CD8^+^ T cells. Antigen presenting cells (APCs), including dendritic cells (DCs), Kupffer cells, liver sinusoidal endothelial cells, and hepatic stellate cells (HSCs), also reside in liver sinusoids. As the hepatic immune system is constantly exposed to harmless dietary and commensal antigens, the liver is often regarded as a tolerogenic organ that favors peripheral tolerance induction [2, 3]. This tolerance is observed under certain conditions, such as administration of antigens *via* the portal vein, allogeneic liver transplantation and certain pathogen infections [4-6]. However, the underlying mechanisms of liver tolerance remain poorly understood.

A variety of immune cells, including NK cells, NKT cells, Kupffer cells, HSCs, and regulatory T cells (Tregs), are involved in the generation of hepatic tolerance [7-13]. As a bridge connecting innate and adaptive immunity, DCs also contribute to immune tolerance through both Treg induction and inhibition of T cell response [14, 15]. These immune tolerance-promoting “regulatory” DCs (DCregs) are derived from immature DCs (imDCs) or redifferentiated mature DCs (mDCs) [16, 17]. Recent findings indicated that liver DCs are characterized by IL-10 secretion [18, 19], and contribute to tolerance maintenance in auto- and allo-immunity models [20, 21]. Subsequent studies demonstrated the presence of liver DCregs, whose generation depended on the liver microenvironment [22-24]. Liver DCregs inhibit CD4^+^ T cell proliferation, direct Th2 response, and induce Tregs [24-27]. However, little is known about liver DCreg regulation of CD8^+^ T cells. As an adaptive immune system component, CD8^+^ T cells play key roles in hepatitis viral clearance, and exert destructive functions in autoimmune hepatitis and during chronic HBV and HCV infection [28, 29]. Understanding how liver DCregs regulate CD8^+^ T cells will enhance comprehension of liver immune tolerance.

In this study, liver stromal cells (LSCs) were used to mimic the liver microenvironment as described previously [24]. We found that LSC-educated mature DCs (LSed-DCs) exhibited increased IL-10 expression and reduced expression of class II MHC molecules and costimulatory molecules. These LSed-DCs acquired the ability to activate CD8^+^ T cells, but inhibited their proliferation, which was associated with enhanced nitric oxide (NO) production. In a CD8^+^ T cell-mediated autoimmune hepatitis (AIH) model, LSed-DCs protected liver against inflammatory damage. This study demonstrated that the liver stroma induces mature DCs to differentiate into regulatory DCs that suppress CD8^+^ T cell proliferation, thus contributing to liver tolerance.

## RESULTS

### Incubation with LSCs induced mDC proliferation

**Figure 1:**
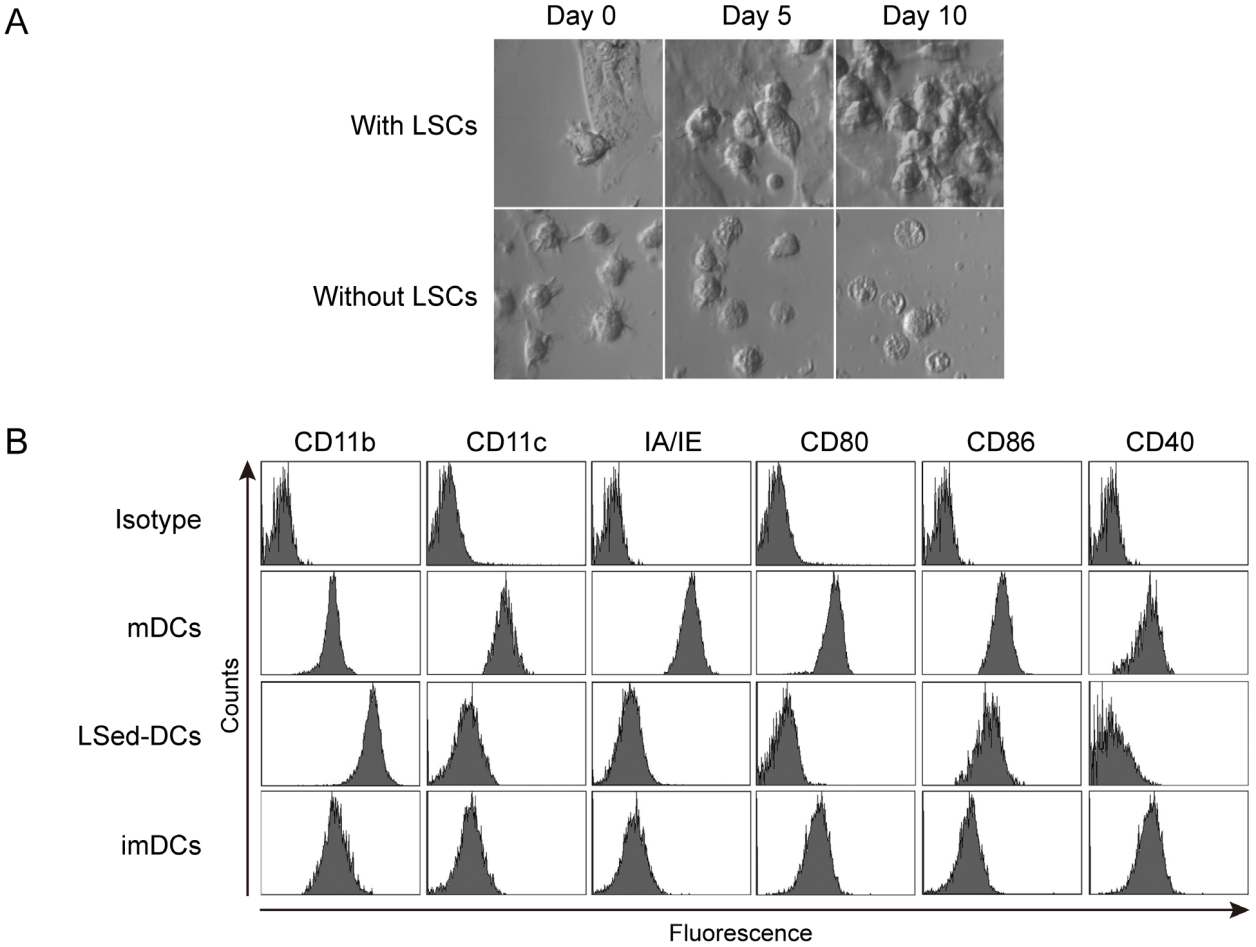
LSed-DC morphology and phenotype Purified BM-derived mDCs were seeded onto LSC monolayers at 2×10^6^ cells/well in 6-well plates. mDC morphology was monitored by phase-contrast microscopy (400×) **A.** After two weeks of incubation with LSCs, LSed-DCs were detected by flow cytometry **B.** BM-derived mDCs and imDCs were used as controls. Data are representative of at least three independent experiments.

To investigate whether the liver microenvironment affected DC differentiation, bone marrow (BM)-derived mDCs from C57BL/6 mice were seeded onto a monolayer of LSCs from CD45.1^+^ B6.SJL mice *in vitro*. After incubation, mDC morphology and expansion were monitored *via* microscopy. Our data showed that mDCs first adhered to the LSCs and subsequently divided into a clone of daughter cells that clustered on the liver stroma monolayer (Figure 1A). Without the support of LSCs, mDCs did not divide and gradually underwent cell death, during which dendrites were lost and intracellular vacuoles appeared (Figure 1A). These data indicated that LSCs could potentially induce mDC proliferation. We further investigated the CD45.1^-^ LSed-DC, mDC, and imDC phenotypes using flow cytometry. LSed-DCs upregulated CD11b, but downregulated CD11c, IA/IE, CD80, CD86, and CD40 as compared to mDCs (Figure 1B). LSed-DCs displayed a phenotype similar to imDCs (Figure 1B). These data indicated that LSCs could educate mDCs. And mDCs displayed plastic potential even at maturation, just like previous findings [16, 30]. However, it should be noted that mDC used here are bone marrow-derived culture-generated mDCs *in vitro*, which may be different from DCs *in vivo*.

### LSed-DCs activated CD8^+^ T cells

**Figure 2:**
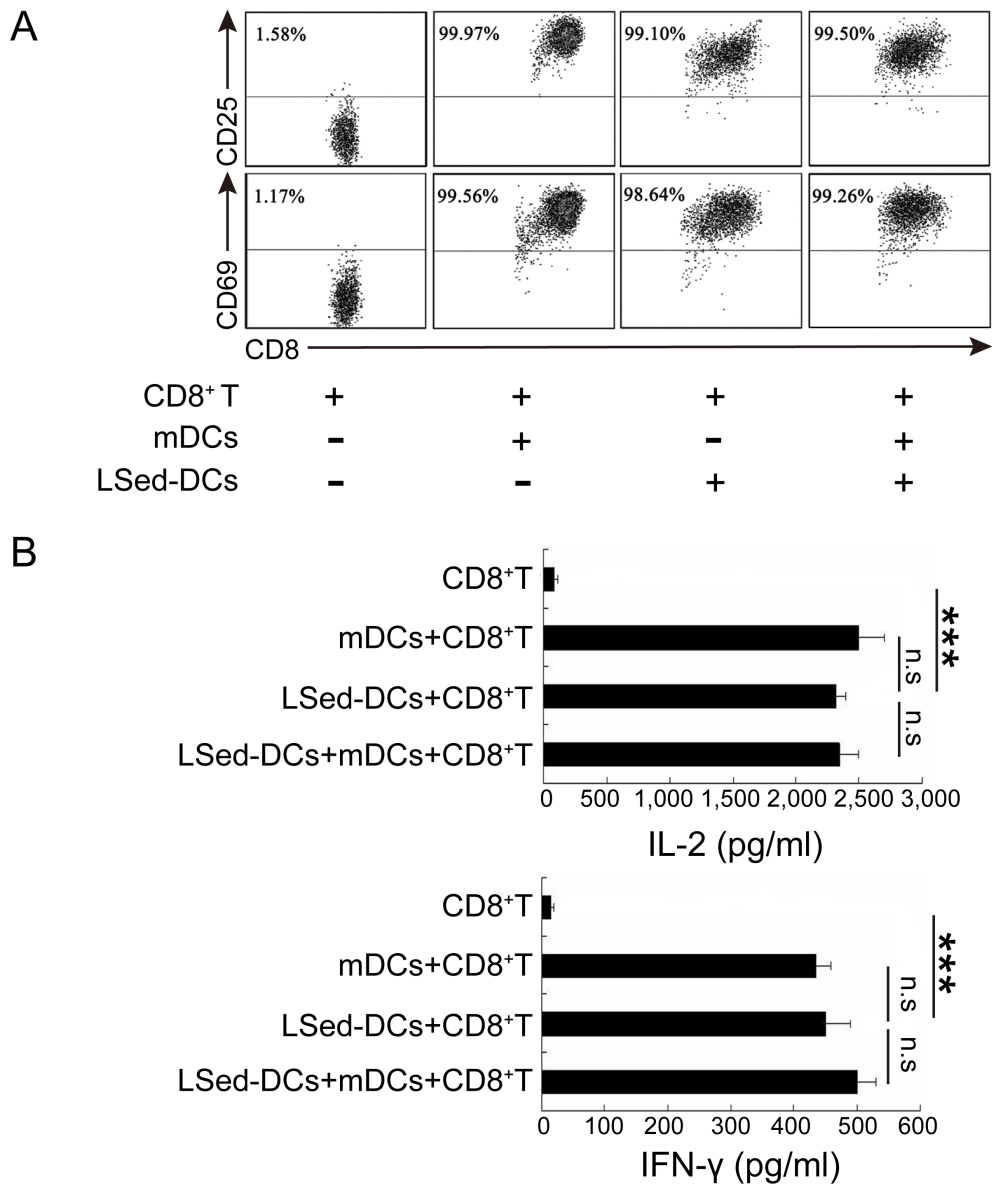
CD8^+^ T cell activation by LSed-DCs Purified OT-1CD8^+^ T cells (2×10^5^) were co-cultured with OVA_257-264_-loaded mDCs (2×10^4^) and/or OVA_257-264_-loaded LSed-DCs (2×10^4^) for 48 h. Cells were collected and CD3^+^/CD8^+^ T cells were gated for analysis of CD25 and CD69 expression by flow cytometry **A.** Co-culture supernatants were collected for analysis of IL-2 and IFN-γ *via* ELISA **B.** Data are presented as means±SD of triplicate wells, and represent three independent experiments. ***P < 0.001, ANOVA.

Considering their unique phenotype, LSed-DCs might direct a different T cell response than that of mDCs. We detected the ability of LSed-DCs to activate CD8^+^ T cells. Naïve OT-I CD8^+^ T cells did not express CD25 and CD69, two markers of T cell activation (Figure 2A). However, > 90% of CD8^+^ T cells expressed CD25 and CD69 when stimulated by OVA_257-264_-loaded mDCs for 48 h. OVA_257-264_-loaded LSed-DCs also promoted CD25 and CD69 expression in CD8^+^ T cells, despite their reduced expression of antigen presentation-associated surface functional molecules. To further confirm CD8^+^ T cell activation, supernatants from co-culture systems were selected for IL-2 and IFN-γ detection. LSed-DC-stimulated CD8^+^ T cells secreted a large amount of IFN-γ and IL-2 when compared with naïve CD8^+^ T cells (Figure 2B). This phenomenon was similar to that observed in co-cultures, including mDCs, or mDCs plus LSed-DCs. Thus, our data indicated that LSed-DCs, in spite of phenotypic alteration, retained the ability to activate CD8^+^ T cells.

### LSed-DCs inhibited CD8^+^ T cell proliferation

**Figure 3:**
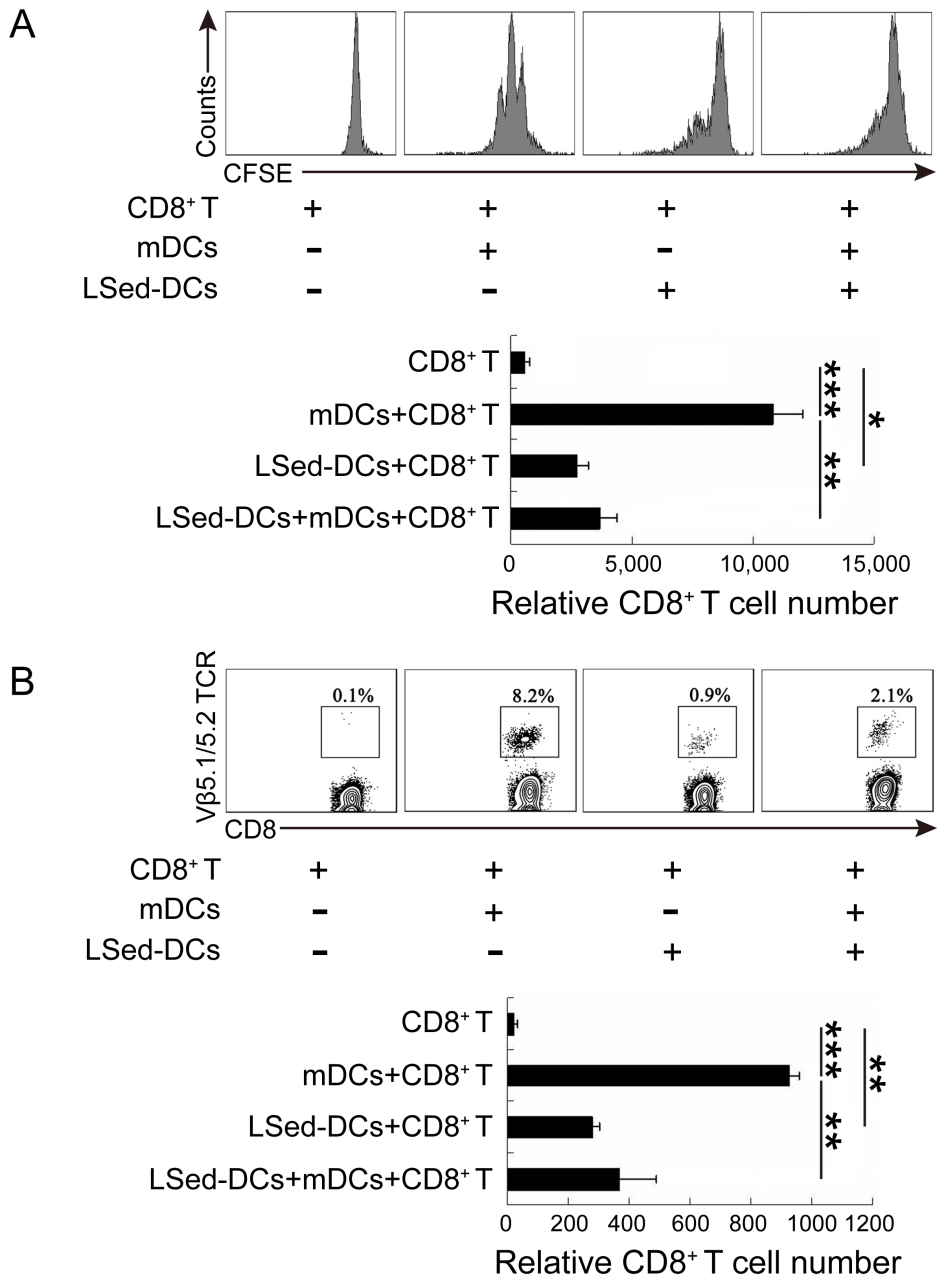
CD8^+^ T cell suppression by LSed-DCs *in vitro* and *in vivo* CFSE-labeled OT-1 splenic CD8^+^ T cells (2×10^5^) were co-cultured with OVA_257-264_-loaded mDCs (2×10^4^) in the presence or absence of LSed-DCs (2×10^4^) for 48 h *in vitro*. CFSE was analyzed in gated CD3^+^/CD8^+^ T cells, and histograms showed relative CD8^+^ T cell numbers as counted by flow cytometry **A.** OT-1 CD8^+^ T cells (1.5×10^6^) with OVA_257-264_-loaded mDCs (1.5×10^5^) and/or LSed-DCs (1.5×10^5^) were transferred intravenously into naive C57BL/6 mice for three days. Mononuclear cells from blood were separated and stained for analysis of OT-1 CD8^+^ T cell (CD3^+^/CD8^+^/Vβ5.1/5.2 TCR^+^) frequency **B.** Histograms showed relative transferred OT-1 CD8^+^ T cell numbers as counted by flow cytometry. Data are presented as means±SD of triplicate wells, and are representative of at least two independent experiments. *P < 0.05, **P < 0.01, ***P < 0.001, ANOVA.

Although LSed-DCs could activate CD8^+^ T cells, weak expression of costimulatory molecules and class II MHC molecules suggested a unique regulatory function for these DCs. We performed a proliferation assay using our co-culture system, with CFSE-labeled OT-1 CD8^+^ T cells and OVA_257-264_-loaded mDCs in the presence or absence of LSed-DCs for 48 h. Flow cytometric analysis showed that mDCs induced repeated division in antigen-specific CD8^+^ T cells, while LSed-DCs weakly promoted OT-1 CD8^+^ T cell proliferation (Figure 3A). Importantly, addition of LSed-DCs impaired mDC-triggered CD8^+^ T cell proliferation. This indicated LSed-DC-mediated suppression, which was supported by decreased CD8^+^ T cell numbers in the mDCs/CD8^+^ T group as compared to the LSed-DCs/mDCs/CD8^+^ T group (Figure 3A). To confirm this LSed-DC inhibitory activity *in vivo*, OT-1 CD8^+^ T cells in combination with OVA_257-264_-loaded mDCs and/or LSed-DCs were transferred into naive C57BL/6 mice. CD8^+^ T cell percentages were detected three days later. We observed a higher percentage of antigen-specific CD8^+^ T cells in blood in the presence of antigen-loaded mDCs. However, LSed-DCs only weakly support OT-I CD8^+^ T cell maintenance. We noted that the percentage of OT-I CD8^+^ T cells in blood decreased when mDCs and LSed-DCs were transferred together, as compared to mDCs alone (Figure 3B). These results matched OT-1 CD8^+^ T cell number changes in blood among the different groups (Figure 3B).

### LSed-DC suppressive activity was not associated with IL-10

**Figure 4:**
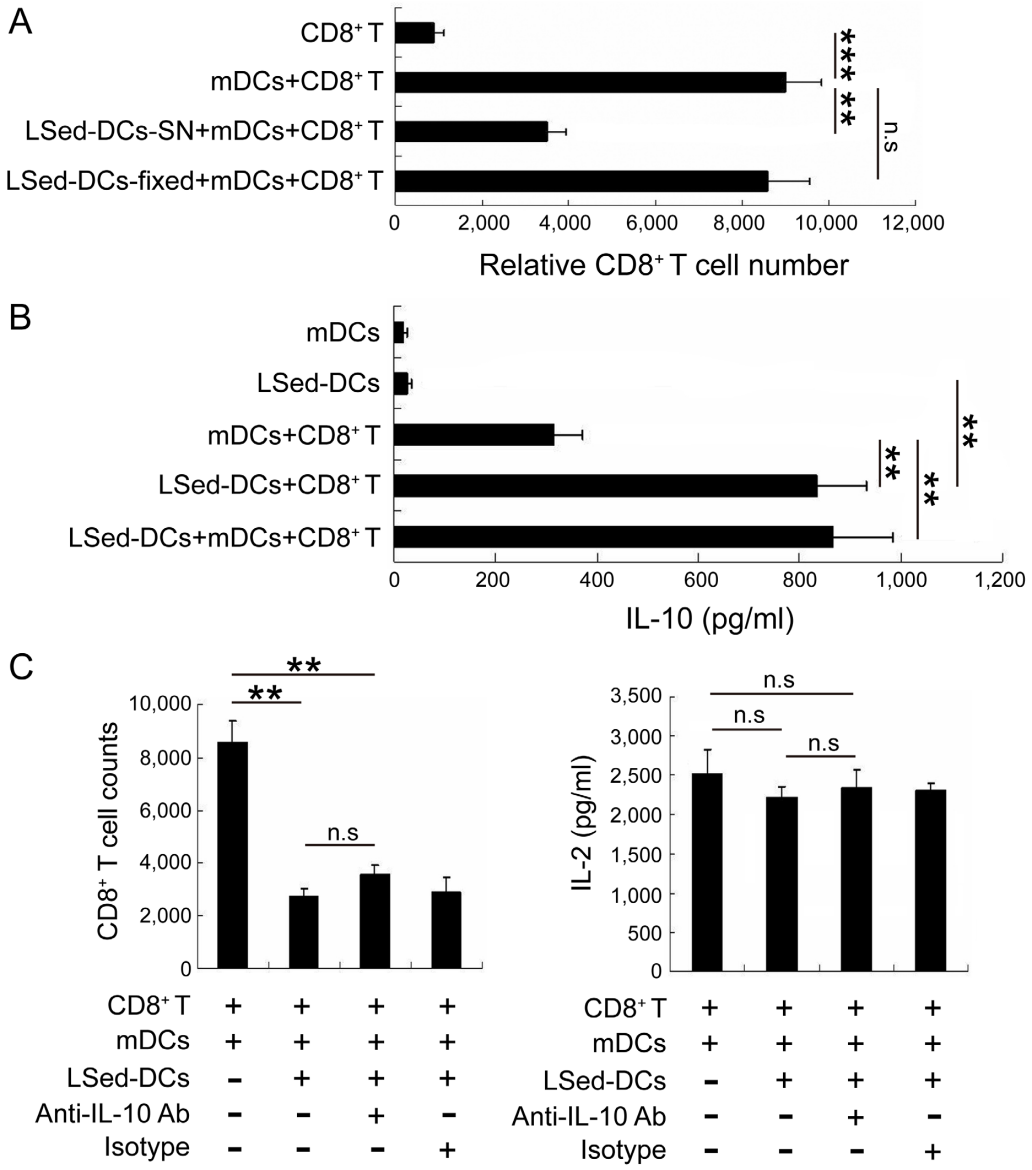
The role of IL-10 in LSed-DC-mediated CD8^+^ T cell suppression Purified LSed-DCs (2×10^4^) were cultured for 48 h. CD8^+^ T cell proliferation was assessed following stimulation with paraformaldehyde-fixed LSed-DCs or LSed-DC culture supernatants **A.** Supernatants from cultures containing mDCs or LSed-DCs were collected at 48 h for analysis of IL-10 *via* ELISA **B.** Anti-IL-10 mAb and matched isotype were added to co-cultures containing LSed-DCs for 48 h, and relative CD8^+^ T cell numbers and IL-2 production were examined by flow cytometry and ELISA **C.** Data are presented as means±SD of triplicate wells, and represent three independent experiments. **P < 0.01, ***P < 0.001 ANOVA.

To assess LSed-DC suppressive mechanisms, we stimulated CD8^+^ T cells with polyformaldehyde-fixed LSed-DCs or LSed-DC culture supernatants. LSed-DC culture supernatants efficiently suppressed CD8^+^ T cell proliferation ,whereas fixed LSed-DCs did so only weakly (Figure 4A). This suggested that soluble factors rather than cell-cell contact might contribute to LSed-DC-mediated CD8^+^ T cell suppression. Additionally, we found that co-culturing DCs and CD8^+^ T cells increased IL-10 production. IL-10 was particularly increased following LSed-DCs/CD8^+^ T and LSed-DCs/mDCs/CD8^+^ T co-cultures, as compared to the mDCs/CD8^+^ T group (Figure 4B). These data suggested that IL-10 might be involved in LSed-DC-mediated CD8^+^ T cell suppression. A blocking experiment with anti-IL-10 mAb was subsequently performed to assess the influence of this cytokine on LSed-DCs-mediated suppression. IL-10 neutralization affected neither CD8^+^ T cell numbers, nor IL-2 production in LSed-DC and mDC co-cultures (Figure 4C). Thus, other soluble factors may contribute to LSed-DC inhibitory activities.

### NO was involved in LSed-DCs-mediated CD8^+^ T cell suppression

**Figure 5:**
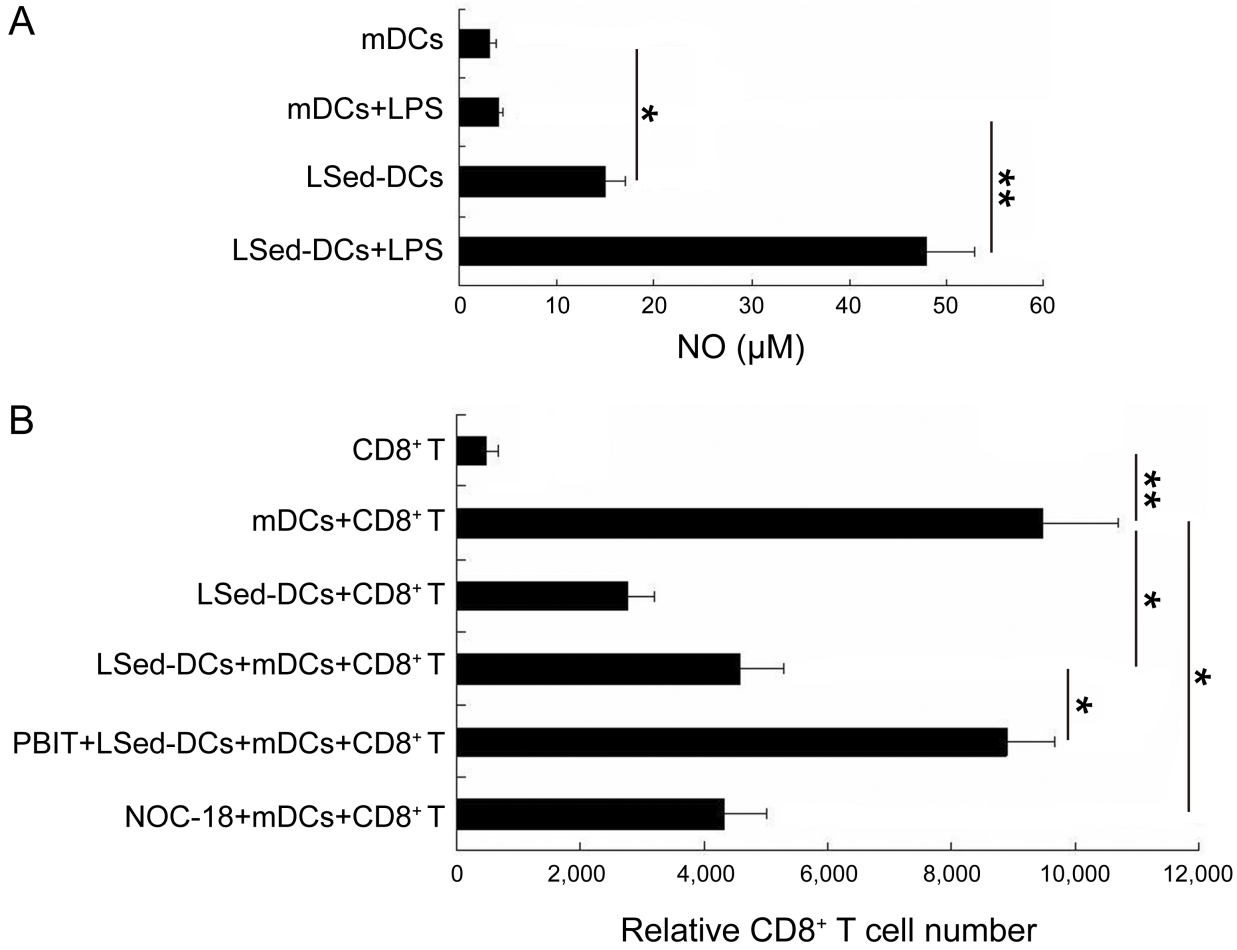
The role of NO in LSed-DC-mediated CD8^+^ T cell suppression Supernatants from cultures containing mDCs (2×10^4^), or LSed-DCs (2×10^4^) with or without LPS (0.5 μg/mL) were collected at 24 h for analysis of NO *via* Griess assay **A.** The NO donor, NOC-18 (10 μM), was added to mDC/CD8^+^ T cell co-cultures, and the NOS inhibitor, PBIT (10μM), was added to LSed-DC/mDC/CD8^+^ T cell co-cultures. 48 h later, relative CD8^+^ T cell numbers were examined using flow cytometry **B.** Data are presented as means±SD of triplicate wells, and represent three independent experiments. *P < 0.05, **P < 0.01, ANOVA.

Previous reports indicated that tissue stroma-educated DCregs suppressed CD4^+^ T cells *via* NO production [16, 17, 31]. We assessed whether NO promoted LSed-DC-mediated CD8^+^ T cell suppression. A Griess assay to detect NO production in different co-culture systems revealed that LSed-DCs produced more NO than mDCs (Figure 5A). Additionally, LSed-DCs rather than mDCs had a greater potential to secrete NO in response to lipopolysaccharide (LPS) stimulation. The data suggested that LSed-DCs might suppress CD8^+^ T cells in a NO-dependent manner. We performed a second proliferation assay in which NO production was either promoted or blocked. Addition of NO donor, NOC-18, to the mDCs/CD8^+^ T co-culture system reduced CD8^+^ T cell numbers, suggesting inhibition by NO (Figure 5B). Furthermore, administration of selective NO synthase inhibitor, PBIT, effectively reversed LSed-DC-mediated CD8^+^ T cell suppression. These data demonstrated that LSed-DCs inhibited CD8^+^ T cell proliferation in a NO-dependent manner.

### LSed-DCs ameliorated CD8^+^ T cell-mediated autoimmune hepatitis

**Figure 6:**
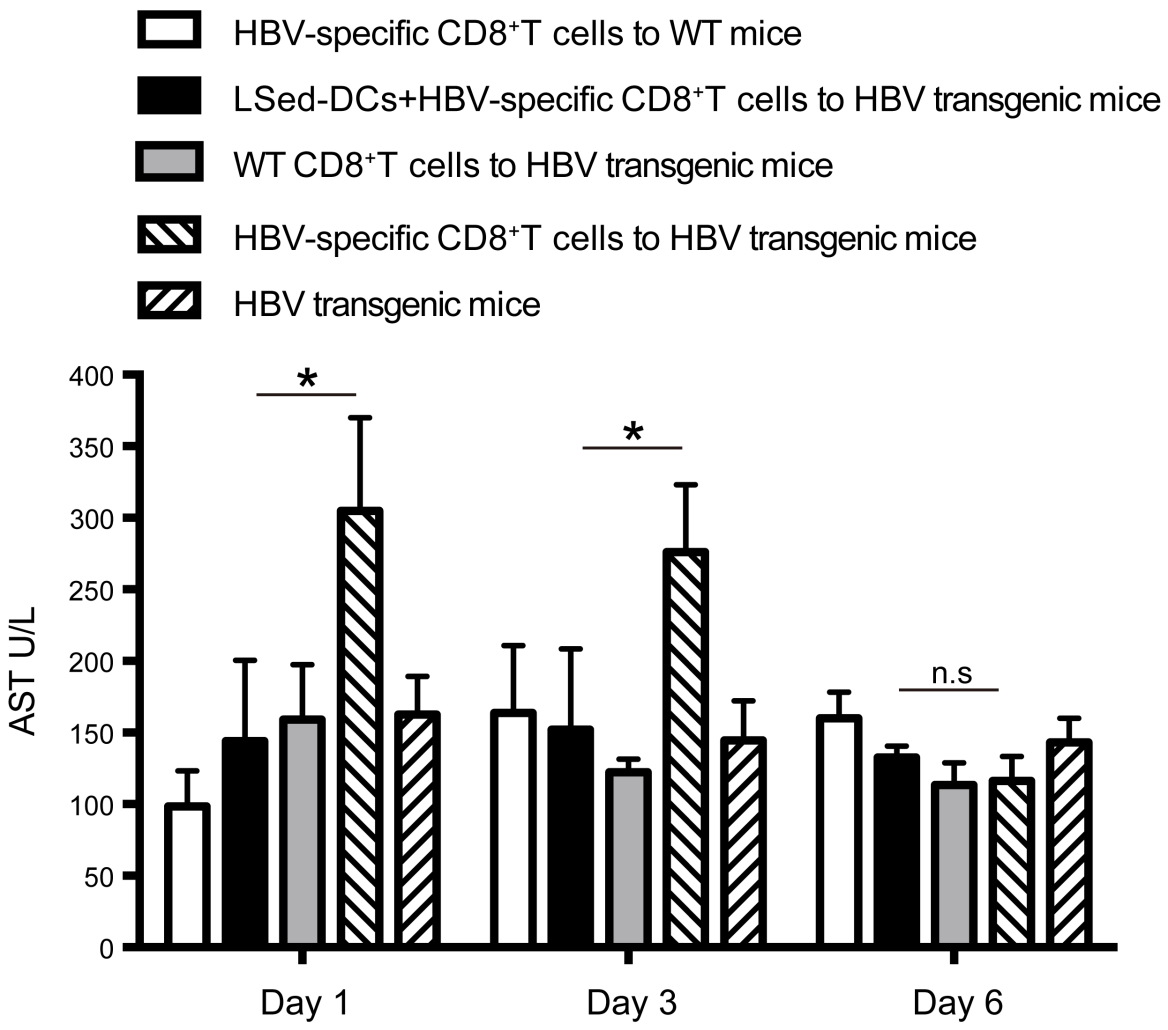
Suppression of AIH by liver LSed-DCs AIH was induced by transfer of HBV-specific CD8^+^ T cells (1×10^8^) into HBV transgenic mice. LSed-DCs (2×10^7^) were also transferred together with HBV-specific CD8^+^ T cells into HBV transgenic mice. HBV transgenic mice receiving CD8^+^ T cells from WT mice or no CD8^+^ T cells, and WT mice receiving HBV-specific CD8^+^ T cells were used as negative controls. Serum AST was detected at different time points. Data are presented as means±SD of triplicate wells, and represent at least two separate experiments. *P < 0.05, ANOVA.

As noted above, LSed-DCs inhibited CD8^+^ T cell proliferation. We hypothesized that these unique LSed-DCs might protect mice from CD8^+^ T cell-mediated liver damage. AIH was induced in mice by transferring HBV-specific CD8^+^ T cells into HBV transgenic mice. Serum AST levels were used to evaluate degree of liver damage. AIH induction increased AST levels from d 1 to 3, followed by recovery on d 6 (Figure 6). This reflected acute hepatitis. However, transfusion of HBV-specific CD8^+^ T cells together with LSed-DCs decreased serum AST on d 1 and d 3, indicating a protective effect by LSed-DCs.

## DISCUSSION

The present study showed that LSCs educated mDCs to differentiate into a unique DC type capable of activating CD8^+^ T cells and inhibiting their proliferation. These LSed-DCs were thought to be a type of DCreg, with a phenotype similar to that of imDCs. Enhanced NO, but not IL-10 expression was associated with LSed-DC-induced CD8^+^ T cell suppression. Additionally, protection of mice against AIH-related tissue damage suggested that LSed-DCs contribute to maintenance of liver tolerance.

The liver is a lymphoid organ that induces tolerance rather than protective immunity [32]. This is due to its unique location that allows for influx of food and other non-pathogen derived antigenic molecules that are absorbed by the intestine. The liver therefore constitutes a set of inflammation-resistant mechanisms that finely regulate the immune response to innocent antigens [2]. Among these mechanisms, liver stroma contributes to the establishment of liver tolerance [33], but how stromal cells exert suppression remains poorly understood. Here, we found that the liver stroma programs mDCs to proliferate and differentiate into DCregs, inducing phenotypic changes as described previously [16, 34, 35]. Our data demonstrated that DCs have plastic potential even at maturation, which was consistent with previous reports [16, 30, 36]. Functional transformation of DCs from the initiation of immunity to the induction of tolerance was also observed in other tissue microenvironments, with diverse underlying mechanisms. For example, both cell-cell contact and soluble factors contributed to the generation of DCregs from mDCs seeded onto splenic stroma, liver stroma, and mesenchymal stem cells [16, 24, 30], while pulmonary stroma and lung cancer cells induced this differentiation in a soluble factor-dependent manner only [17, 31].

In agreement with previous findings, liver DCregs suppress CD4^+^ T cell proliferation and induce apoptosis of the activated CD4^+^ T cells [14, 24, 37]. However, information about DCreg-induced CD8^+^ T cell inhibition was lacking. We found that LSed-DCs retained the ability to activate CD8^+^ T cells, but inhibited mDC-triggered CD8^+^ T cell proliferation. Inhibition of antigen-specific CD8^+^ T cells may help the liver recover from autoimmune disease, and our results support this hypothesis.

DCregs can cause immunosuppression by a variety of mechanisms [38-41]. We found here that LSed-DC-secreted soluble factors, but not cell-cell contact, contributed to CD8^+^ T cell proliferation. Previous reports documented that human liver DCs require IL-10 to generate CD4^+^/CD25^+^/Foxp3^+^ Tregs [27]. Mouse liver DCs reduced TNF, IL-6, and ROS production by inflammatory monocytes through IL-10 secretion [18]. These data suggested the importance of IL-10 in liver DC-mediated suppression. Although we found that LSed-DCs produced more IL-10 than mDCs, our results showed that LSed-DCs suppressed CD8^+^ T cells in IL-10-independent manner. However, suppression of CD4^+^ T cells by DCregs *via* NO is well recognized [16, 17, 31, 42], and we found that LSed-DCs suppressed CD8^+^ T cells in NO-dependent manner. NO effects on CD8^+^ T cells must be further investigated.

Together, the data presented in this study showed that LSCs induced mDC differentiation into DCregs, which suppressed CD8^+^ T cell proliferation. Our results support the hypothesis that mDCs differentiate into DCregs after exposure to the stromal environment, rather than undergoing activation-induced apoptosis, and enhance our understanding of the liver microenvironment in liver tolerance and immune homeostasis.

## MATERIALS AND METHODS

### Mice

C57BL/6 mice, CD45.1^+^ B6 SJL mice, OVA_257-264_ specific TCR transgenic mice (OT-1 mice), and HBV transgenic mice, all 6-8 weeks of age, were purchased from the animal facility at Tsinghua University, China. Studies were approved by the Laboratory Animal Care Committee of Tsinghua University, and all animal experiments were conducted in accordance with the Guidelines of Care and Use of Laboratory Animals at Tsinghua University.

### LSC culture

LSCs were prepared as described previously [24]. Briefly, the liver was prepared from newborn B6 SJL (CD45.1^+^) mice, cut into 1-2mm (length) fragments, and cultured in RPMI 1640 medium supplemented with 20% fetal calf serum (FCS; PAA Laboratory, Pasching, Austria) at 37° with 5% CO_2_. After 2-3 weeks, relatively fast-growing cells were harvested and purified using CD11b microbeads (Miltenyi Biotec, Auburn, CA) to remove contaminating liver macrophages. Such cells, designated LSCs, were characterized by positive vimentin, desmin, and α-smooth muscle actin expression, and negative cytokeratin-7, CD105, and CD54 expression. They displayed fibroblast morphology, and were expanded for use in the following experiments.

### Preparation of mDCs from mouse bone marrow

mDCs were prepared from BM progenitors as described previously [16]. Briefly, BM mononuclear cells were prepared from mouse femur BM suspensions and cultured in RPMI 1640 medium containing 10% FCS, 10 ng/ml of recombinant mouse granulocyte macrophage colony-stimulating factor (GM-CSF), and 1 ng/ml of recombinant mouse IL-4 (PeproTec, London, United Kingdom). Nonadherent cells were gently washed out on d 4; the remaining loosely adherent clusters were further cultured in the presence (used as mDCs) or absence (used as imDCs) of 10 ng/ml of LPS (Sigma-Aldrich) for 4 d, and then positively sorted with CD11c magnetic microbeads (Miltenyi Biotec). DC purity was tyity of tively soby flow cytometry. In some experiments, prepared mDCs were cultured for 48 h, and supernatants were collected for cytokine analysis *via* ELISA.

### mDC education by LSCs

mDCs derived from C57BL/6 mice were seeded onto LSC monolayers derived from CD45.1^+^ B6 SJL mice in 6-well plates at 2×10^6^ cells in 5ml per well, then the media was replaced by RPMI 1640 medium supplemented with 5% FCS to suppress stromal cell growth. After co-culture with LSCs for two weeks, educated cells (viability≥90%) were washed off the monolayer with 0.1% trypsin and 5mM EDTA, and CD45.2^+^/CD45.1^-^/CD11c^+^ mDCs (LSed-DCs) were purified by flow cytometry (purity≥90%). mDCs and LSCs were derived from different species and had different markers, which helped to avoid LSC contamination during educated mDC sorting. LSed-DC numbers, morphologies and phenotypes were examined *via* phase-contrast microscopy (Leica-DMIRB; Leica, Wetzlar, Germany) and flow cytometry. In some experiments, prepared LSed-DCs were cultured for 48 h, and supernatants were collected for cytokine analysis *via* ELISA.

### Antibodies and flow cytometry

Fluorescein-conjugated mAbs specific for the mouse antigens, CD3 (145-2C11), CD8 (53-6.7), CD11b (M1/70), CD11c (N418), IA/IE (MKS4), CD80 (16-10A1), CD86 (GL1), CD40 (1C10), CD25 (PC61.5), CD69 (H1.2F3), Vβ5.1/5.2 (MR9-4), CD45.1 (A20), and CD45.2 (104) were purchased from eBioscience (San Diego, CA). An IL-10 neutralizing mAb was purchased from R&D Systems (Minneapolis, MN). For cell surface staining, cells were incubated with fluorescence-conjugated mAbs in the presence of 2.4G2 or rat sera. Matched isotype controls were used to establish background fluorescence. 7-AAD was used to exclude dead cells in phenotype analysis. For cell counting, stained cells were collected at high speed for 40 sec and counted *via* flow cytometer. Phenotype analysis and cell counting were performed on a BD FACSAria (BD Biosciences, Sand Jose, CA) using the BD FACSDiVa software (BD Biosciences).

### CD8^+^ T cell proliferation assay

For *in vitro* assays, CD3^+^/CD8^+^ T cells (purity rolifwere obtained using flow cytometry sorting from spleens of naive OT-1 mice, and labeled with 5 μM CFSE (Molecular Probes, Eugene, OR). 2×10^5^ CD8^+^ T cells were then co-cultured with 2×10^4^ OVA_257-264_-loaded mDCs (co-culture with 2 mM OVA_257-264_ peptide at 37°C for 6 h) in 96-well plates with or without 2×10^4^ LSed-DCs, 2×10^4^ paraformaldehyde-fixed LSed-DCs, or LSed-DC supernatants (LSed-DCs-SN, collected after 48 h culture). After 48 h, cells were stained with anti-CD8 mAb followed by proliferation analysis using flow cytometry.

For *in vivo* assays, 1.5×10^6^ OT-1 CD8^+^ T cells, 1.5×10^5^ OVA_257-264_-loaded mDCs, and 1.5×10^5^ LSed-DCs were injected intravenously into C57BL/6 mice. Three d later, mononuclear cell suspensions were prepared from the blood of recipient mice to determine the percentage of transferred OT-1 CD8^+^ T cells (CD3^+^/CD8^+^/Vβ5.1/5.2 TCR^+^) *via* flow cytometry. In certain proliferation assays, anti-IL-10 mAb (20 μg/ml), the selective NO synthase inhibitor PBIT (10 μM), or the NO donor NOC-18 (10 μM) were administered.

### Cytokines and NO assay

2×10^5^ OT-1 CD8^+^ T cells were co-cultured with 2×10^4^ OVA_257-264_-loaded mDCs in 96-well plates with or without the same number of liver LSed-DCs. 48 h later, IL-2, IFN-γ, and IL-10 levels in co-culture supernatants were determined using the Ready-SET-Go ELISA Kit (eBioscience, San Diego, CA, USA) according to the manufacturer’s instructions. For NO detection, mDCs and LSed-DCs were cultured for 24 h with or without LPS (0.5 μg/mL). NO production was determined by measuring nitrite concentration using the Griess assay as described previously [43].

### Preparation of AIH model

Wild type (WT) C57BL/6 mice were immunized *via* intraperitoneal injection of 1×10^8^ liver cells from HBV transgenic mice. Three boosts with 5×10^7^ HBV-transgenic liver cells each were performed at 10-d intervals after initial challenge. 45 d after initial immunization, splenic CD8^+^ T cells (1×10^8^) were sorted (purityorted as HBV-specific CD8^+^ T cells using CD8^+^ magnetic microbeads (Miltenyi Biotec), and injected intravenously into HBV transgenic mice to induce AIH. Degree of liver damage was examined *via* aspartate aminotransferase (AST) detection in blood on d 1, 3 and 6. HBV transgenic mice receiving CD8^+^ T cells from WT mice or no CD8^+^ T cells, and WT mice receiving HBV-specific CD8^+^ T cells were used as negative controls. In the AIH model, 2×10^7^ LSed-DCs were injected intravenously together with HBV-specific CD8^+^ T cells to investigate LSed-DC-related protection against autoimmune damage.

### Statistical analysis

Significant differences were assessed using Student’s *t* tests for two groups or ANOVA for 3-4 groups. *P* < 0.05 was considered significantly different.
